# Incidence and risk of necrotizing enterocolitis in Denmark from 1994-2014

**DOI:** 10.1371/journal.pone.0219268

**Published:** 2019-07-08

**Authors:** Sandra Meinich Juhl, Rasmus Gregersen, Theis Lange, Gorm Greisen

**Affiliations:** 1 Department of Neonatology, Rigshospitalet, Copenhagen, Denmark; 2 Section of Biostatistics, University of Copenhagen, Copenhagen, Denmark; 3 Center for Statistical Science, Peking University, Beijing, China; Centre Hospitalier Universitaire Vaudois, FRANCE

## Abstract

Introduction. We suspected that the incidence of NEC in Denmark had increased during the last 20 years but hypothesized that this could be explained by the increased neonatal survival. Methods. We conducted a retrospective, observational cohort study of all registered liveborn infants in Denmark in the period from January 1, 1994 to December 31, 2014. Data were obtained from the Medical Birth Registry, National Patient Register, and Cause of Death register in Denmark. The primary outcome was the registration of NEC (ICD-10: DP77.9) during a hospital admission within 6 months after birth. The statistical analysis used ‘death before NEC’ as a competing risk. Results. The cohort consisted of 1,351,675 infants, of which 8,059 died. There was a strongly significant decreasing risk of death over the period for the all infants (p<0.0001 in all gestational age groups). In total, 994 infants were diagnosed with NEC which lead to an incidence of 7.4 per 10,000 live-born infants. During the observation period, the incidence increased from 6.3 to 7.9 per 10,000 births (p = 0.006). When accounting for ‘death before NEC’ as a competing risk, the increase could be explained by the increased neonatal survival. There was, however, a GA-group/epoch interaction (p = 0.008) in the cause-specific hazard ratios with a trend towards an increasing risk of NEC in the most preterm infants and a decreasing risk of NEC in the term infants. Conclusion. While the overall incidence of NEC increased over the study period, the overall risk of NEC did not increase when considering the increased survival. Nevertheless, there seemed to be an increased risk of NEC in the most premature infants which was masked by a decreased risk in the term infants. This study suggests that research to prevent NEC in the most preterm infants is more important now than ever.

## Introduction

Necrotizing enterocolitis (NEC) is a gastrointestinal emergency, most often seen in preterm infants [1]. The risk of developing NEC increases exponentially with the degree of immaturity [2]. The mortality of extremely preterm infants has decreased during the last 20 years, however the proportion of deaths related to NEC has increased in both the US and UK [3,4]. Furthermore, in a Swedish population-based study of live-born infants from 1987 to 2009, the incidence of NEC increased while the seven-day all-cause mortality decreased, especially in the extremely preterm infants [5]. In the Netherlands, an association was observed between introducing a guideline with more active treatment of extremely preterm infants and an increase in the incidence of NEC [6]. Together, these observations suggest that the observed increase in NEC incidence might be caused by an increase in survival in the first days and weeks of life among the most preterm infants. This would have the effect of exposing more infants to the risk of NEC, which is a disease that typically develop weeks after birth. To investigate this potential relation, it is necessary to use a formal competing risk analysis, where both the risk of NEC and the risk of death are considered concurrently. None of the previous studies investigating NEC incidence have done so.

The incidence of NEC among very low birth weight (VLBW) infants across the world appears to vary between 1 and 10% [7]. In Sweden and UK, no statistically significant variation in incidence was seen among hospitals, except for an increased incidence of NEC in the area of the capital in Sweden, which was, however, not accompanied by an increased 28-day-mortality [5,8]. In Denmark, the national incidence has not previously been studied. Regional data from Western and Eastern Denmark have given quite different estimates of the incidence in VLBW infants, although from very different periods of time [7,9].

We suspected that the incidence of NEC among newborn infants in Denmark has increased during the last 20 years and hypothesized that this trend would disappear when adjusting for the increased survival of premature infants.

## Materials and methods

### Study design, data sources, cohort, and ethics

We conducted a retrospective, observational cohort study of all registered liveborn infants in Denmark in the period from January 1, 1994 to December 31, 2014. ‘Statistics Denmark’ created a database for this study by linking data from the Medical Birth Registry, National Patient Register, and Cause of Death register in Denmark. Identification and linkage were done by using the Central Person Register (CPR)-number which is assigned to every citizen at birth or immigration and thereby enable reliable identification of subjects in all registers. Many aspects of the Danish registers have been described and validated [10–12]. Children born abroad and children with missing data on region of birth were excluded. The study was approved by Statistics Denmark, Copenhagen, Denmark, (e-mail: dst@dst.dk with the project number 706458. According to Danish law, no approval from an ethical committee or the data protection agency was needed. Reporting adhered to RECORD guidelines, an extension to the STROBE statement intended for studies using routinely collected data such as the Danish registers [13].

### Primary outcome

The primary outcome was the registration of NEC (ICD-10: DP77.9) during an admission starting within 180 days (6 months) after birth. The code DP77.9 –*Necrotizing enterocolitis in newborn*, *unspecified*, has been used universally in Denmark for all cases of NEC since 1994 when the the tenth version of the International Classification of Diseases (ICD-10) was introduced. In Denmark, a national ‘Danish Health Authority’ adaptation of the international WHO ICD-10 codes is used, which includes extra, more specific sub-groups. In this study, ICD-10 codes marked with a * indicates that all sub-groups were also included.

### Secondary outcomes

We registered mortality (available until 2015), gastrointestinal surgery, other gastrointestinal morbidities, and sequalae following NEC as secondary outcomes,. We defined the primary admission as the admission during which NEC was diagnosed. Gastrointestinal surgeries were registered by the NOMESCO (The Nordic Medico-Statistical Committee) Classification of Surgical Procedures [14], and were defined as either primary surgery (the first relevant procedure) or re-operation. A gastrointestinal procedure was defined from the procedure-groups KJA (procedures of the abdominal wall, mesentery, peritoneum, and greater omentum), KJF (procedures of the intestines), or KJH (procedures of the anus and perianal tissue), but excluding hernia procedures. Re-operation was defined from the same codes as those of primary surgery and KJW (reoperations in gastroenterological surgery), and defined as occuring after primary surgery but before discharge to home. Furthermore, we registerered enterostoma from relevant procedures in KJF and KJG (procedures of the rectum), either as primary (same day as primary surgery) or secondary (during admission, but later than primary surgery). We also registered if the patient died related to the primary surgery (same or following day).

Gastrointestinal morbidity was registered during primary admission (ICD-10) as ileus (K560*, K562*, K565* or K567*), perforation or peritonitis (DK223*, DK281*-282*, DK628*, DP780*, DK650, DK650M-P), abscess (DK61*, DK630, DK650A-DK650L), or fistula (DK316*, DK603*-604*, DK605, DK632*).

Sequaele following NEC was registered after discarge from primary admission as ileus (see above), short-bowel syndrome (DK912B), stricture or stenosis (DK222C, DK312*, DK315A-B, DK566F-G, DK624*, DK913A-C), and fistula (see above).

### Exposure variables

The exposure variables of interest were gestational age (GA), birth weight (BW), small for gestational age (SGA), year of birth, gender, and region of birth. GA was reported in full weeks and divided into one term group (≥37 w) and three groups of pre-terms (32–36, 28–31, and 23–27 w). BWs were divided into one group of normal BW (≥2500 g) and 5 groups of low BWs (2000–2499, 1500–1999, 1000–1499, 750–999 and <750 g). Under the assumption of mis-registration, we defined GA <23 or >45 weeks or BW <100 g or >9000 g as missing. If expected BW for GA was outside the interval of +/- 5 SD (calculated from Marsal’s formula[15]) both GA and BW were set as missing [15]. SGA was defined as BW <-2 SD from expected BW. Birth year was divided into 4 epochs of 5–6 years (1994–1998, 1999–2003, 2004–2008 and 2009–2014). Surgical NEC treatment is centralized to one unit in eastern Denmark and one unit in western Denmark. Therefore, region of birth was divided into East (Capital Region of Denmark and Region Zealand) and West (Region of North Denmark, Region of Central Denmark, and Region of Southern Denmark).

### Statistical analysis

Main analyses were done using cumulative incidence functions (CIF), with NEC diagnosis and ‘death prior to NEC diagnosis’ as competing risks. With competing risk analyses, results can be adjusted for the possibility that children dying from other causes than NEC could have developed NEC, had they survived longer. This contrasts a naïve analysis that simply counts the number of NEC cases per 10.000 births in a given strata. In Denmark, diagnoses given during an admission are not reported until the patient is discharged from the department, so the precise date of NEC onset was not known. Due to this interval-censoring, the date of NEC diagnosis was defined as the mid of the admission during which NEC was registrered. Incidences were registered as CIF at 180 days, stratified by exposure variables in univariable analyses with significance assessed by Grays Test. The effect of GA and epoch on the risk of NEC was assessed with the cause-specific hazards approach with Cox regression on the competing risk of NEC and death respectively and censoring by the other competing risk. These analyses were done stratified by GA-groups and adjusted for epoch, SGA and gender, and reported as cause-specific hazard rates (CSHR). Analyses for ‘death before NEC’ during admission was done by the Chi-square test. Analysis of sequelae (short-bowel syndrome and the occurrence of strictures and/or fistulas) following NEC was done using cumulative incidence functions with death before sequalae as a competing risk until end of follow-up. Analyses were performed in SAS Statistical Software (Version 9.4, SAS Institute, USA).

## Results

**Fig 1:**
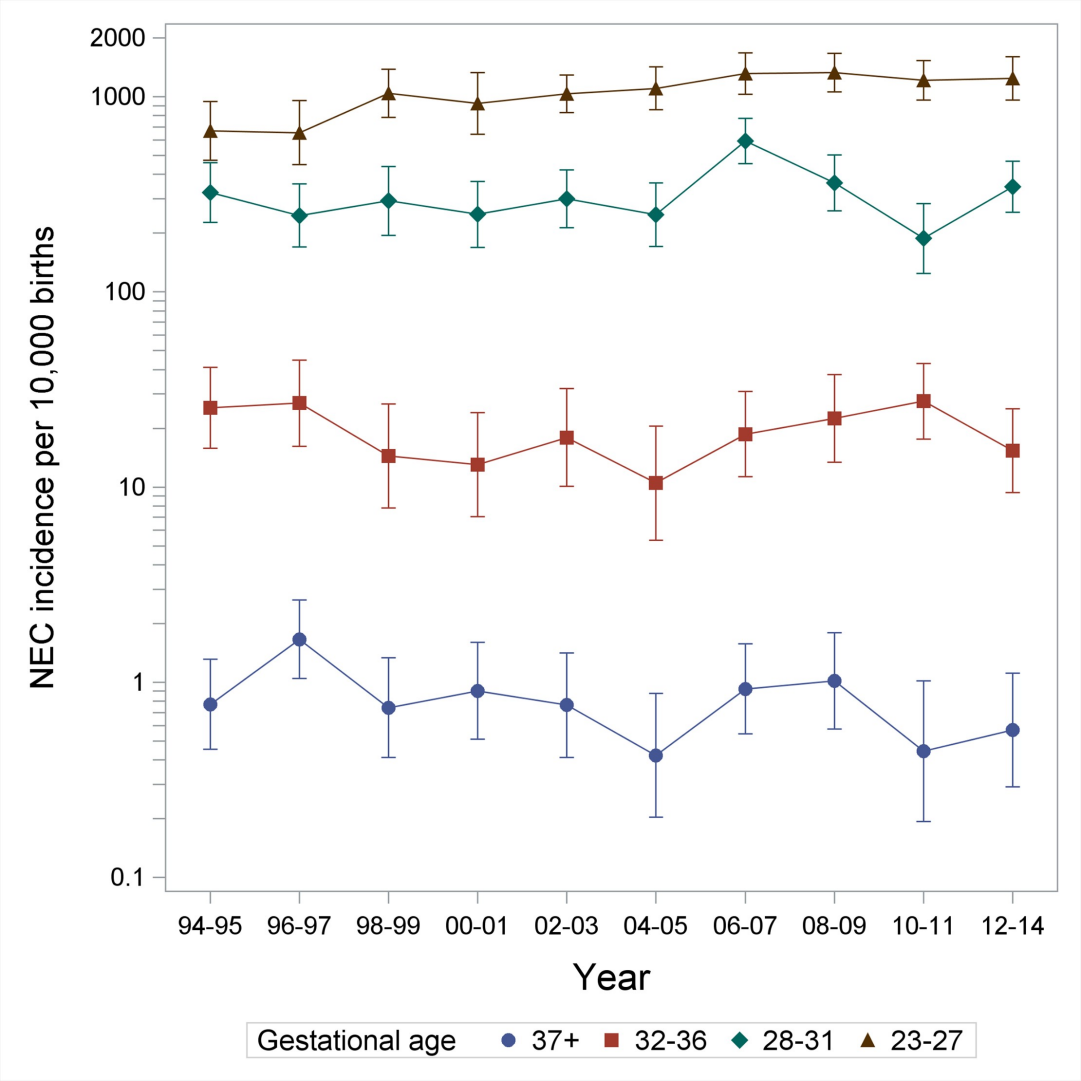
Number of NEC cases per 10,000 children born according to year of birth, divided into four gestational age groups. Please note, that the y-axis is logarithmic.

In total, 1,354,017 live-born children were included in the registries and we included 1,351,675 infants in the study after excluding 471 infants born abroad and 1,871 infants with missing data on region of birth. During the period, 994 infants were given the NEC diagnosis which led to an incidence of 7.4 per 10,000 live-born infants (Table 1). The incidence increased significantly with decreasing GA from 0.82 per 10,000 for term born infants to 1063.2 per 10,000 for infants born before week 28 (p<0.0001). The incidence also increased with decreasing BW (p<0.0001). From the first epoch to the last, the incidence increased from 6.27 to 7.89 per 10,000 births (p = 0.006) (Table 1). The increase in incidence over time was due to an increase among the most premature infants (Fig 1). The overall incidence of NEC did not differ between East- and West Denmark (7.00 vs. 7.76 per 10,000 births, p = 0.10) (Table 1).

**Table 1:** Necrotizing enterocolitis (NEC) incidence calculated as cumulative incidence function (CIF) at 180 days.

	Number of births with available information	NEC cases	Deaths (before NEC) within 180 days of birth	CIF incidence at 180 days (per 10.000 births)	P-value (Gray’s test)
All	1,351,675	994	5089	7.35 (6.96–7.77)	
GA					
≥37 wks	1,237,461	101	1867	0.82 (0.68–0.97)	<0.0001
32–36 wks	73662	139	701	18.9 (15.9–22.4)	
28–31 wks	9756	309	431	316.6 (283.3–353.8)	
<28 wks	3988	424	1283	1063.2 (979.6–1154.0)	
BW					
>2500 g	1260408	114	1808	0.90 (0.76–1.08)	<0.0001
2000–2499 g	42560	43	371	10.1 (7.48–13.6)	
1500–1999 g	15211	102	316	67.0 (55.2–81.5)	
1000–1499 g	7309	251	374	343.3 (302.2–390.0)	
750–999 g	2376	211	414	888.0 (781.5–1009.0)	
<750 g	2346	242	1371	1030.1 (910.9–1164.9)	
SGA					
No	1283107	659	3226	5.14 (4.75–5.55)	<0.0001
Yes	68568	335	1863	48.9 (43.4–55.0)	
Epochs					
1994–1998	341079	213	1536	6.24 (5.42–7.20)	0.006
1999–2003	328410	227	1328	6.91 (6.02–7.93)	
2004–2008	324751	272	1137	8.38 (7.47–9.39)	
2009–2014	357435	282	1088	7.89 (7.08–8.80)	
Gender					
Girls	658219	455	2255	6.91 (6.37–7.51)	0.07
Boys	693456	539	2834	7.77 (7.16–8.44)	
Geographic region					
East Denmark	623375	484	2336	7.76 (7.10–8.49)	0.10
West Denmark	728300	510	2753	7.00 (6.44–7.61)	

CIF calculated isolated for all individuals and univariably by each exposure variable. GA = Gestational age, BW = Birth weight, SGA = small for gestational age.

To account for the effect of increased survival among the extremely preterm infants during the period, we also used a competing risk approach with ‘death before NEC diagnosis’ as a competing risk. Initially, we conducted an unstratified analysis on CSHR for NEC adjusted for GA, epoch, gender, SGA, and the GA * epoch interaction, revealing a significant interaction (p = 0.008). The overall CSHR for NEC did not change significantly over the epochs, when not accounting for GA (p = 0.12, Table 2). To further elaborate on the interaction between GA and epoch, we performed analyses stratified by GA-groups and found a trend towards an increasing risk of NEC for the extremely preterm infants with the cause-specific hazard ratio (CSHR) increasing to 1.46 (1.07–1.98) in 2004–2008 and to 1.39 (1.03–1.88) in 2009–2014, compared to 1994–1998 (p = 0.06) (Table 2). The risk of NEC for the 29-32-week group also tended to increase, while the 33-36-week group and the term group tended to decrease (Table 2). In contrast, the risk of death before NEC decreased consistently over the period for the all four groups of infants (p<0.0001 in all GA-groups) and the relative decrease did not differ substantially between the GA-groups. The mean GA in each GA group did not change markedly over time (Table 2). SGA-children were at a significantly increased risk of developing NEC in univariable analysis (Table 1) which was also significant when adjusting for GA and BW in the competing risk analysis. Being born SGA increased the risk of death (CSHR 3.27 (3.03–3.52)) more than risk of NEC (CSHR 2.76 (2.40–3.17)) even when adjusting for BW and GA. The small difference in incidence observed between genders was statistically insignificant (p = 0.07).

**Table 2:** Effect of GA and epoch on risk of NEC, using a competing risk approach.

GA	Epoch	Mean GA	CSHR for NEC	P-value (type-III test)	CSHR for death	P-value (type-III test)
Analysis adjusted for GA, epoch, SGA, gender						
37+		39.7	1 (ref)	<0.0001	1 (ref)	<0.0001
32–36		34.9	20.6 (15.9–26.6)		5.46 (5.00–5.96)	
28–31		29.8	314.7 (250.2–395.9)		21.9 (19.7–24.4)	
23–27		25.6	1550.5 (1243.1–1993.9)		212.3 (196.9–229.0)	
	1994–1998	39.4	1 (ref)	0.12	1 (ref)	<0.0001
	1999–2003	39.3	0.93 (0.77–1.12)		0.80 (0.74–0.87)	
	2004–2008	39.2	1.14 (0.95–1.37)		0.63 (0.58–0.69)	
	2009–2014	39.2	1.05 (0.88–1.26)		0.47 (0.43–0.51)	
Analyses stratified by GA, adjusted for epoch, SGA, and gender						
37+	1994–1998	39.8	1 (ref.)	0.21	1 (ref)	<0.0001
37+	1999–2003	39.7	0.72 (0.43–1.22)		0.84 (0.75–0.95)	
37+	2004–2008	39.6	0.62 (0.36–1.08)		0.64 (0.56–0.73)	
37+	2009–2014	39.6	0.61 (0.36–1.04)		0.46 (0.40–0.52)	
32–36	1994–1998	34.9	1 (ref.)	0.07	1 (ref)	<0.0001
32–36	1999–2003	34.9	0.60 (0.37–0.98)		0.89 (0.74–1.07)	
32–36	2004–2008	34.9	0.62 (0.39–1.01)		0.55 (0.45–0.68)	
32–36	2009–2014	34.9	0.94 (0.61–1.44)		0.45 (0.36–0.56)	
28–31	1994–1998	29.8	1 (ref.)	0.04	1 (ref)	<0.0001
28–31	1999–2003	29.8	1.02 (0.73–1.44)		0.99 (0.78–1.25)	
28–31	2004–2008	29.8	1.46 (1.07–1.99)		0.58 (0.44–0.76)	
28–31	2009–2014	29.8	1.05 (0.75–1.46)		0.50 (0.38–0.67)	
23–27	1994–1998	25.7	1 (ref.)	0.06	1 (ref)	<0.0001
23–27	1999–2003	25.6	1.18 (0.86–1.61)		0.71 (0.61–0.82)	
23–27	2004–2008	25.5	1.46 (1.07–1.98)		0.75 (0.64–0.87)	
23–27	2009–2014	25.5	1.39 (1.03–1.88)		0.54 (0.46–0.64)	

Analyses both unstratified and stratified by GA. Also adjusted for SGA (small for gestational age) and gender. GA = Gestational age, CSHR = cause-specific hazard ratio

Of the 994 infants with NEC, 290 died before discharge at a median age of 12 days (Table 3). The death rate per 100 NEC cases was 29.2, increasing from 12.9 in infants born after 37 weeks of GA to 44.6 among infants born at 23–27 weeks of GA (p<0.0001). Likewise, the death rate (lethality) increased with decreasing BW. There was no significant difference in the death rate over the four epochs and no association with SGA, gender or region.

**Table 3:** Death during admission for NEC cases.

Characteristic	Nec cases	Number of deaths during admission	Death rate per 100 NEC cases	OR for death	P-value	Median age of death (days)
All	995	290	29.2			12
GA						
≥37 wks	101	13	12.9	1 (ref.)	<0.0001	9
32–36 wks	139	25	18.0	1.48 (0.72–3.07)		7
28–31 wks	309	54	17.5	1.43 (0.75–2.75)		11
23–27 wks	424	189	44.6	5.44 (2.95–10.0)		12
BW						
>2500 g	114	13	11.4	1 (ref.)	<0.0001	50
2000–2499 g	43	8	18.6	1.78 (0.68–4.64)		6
1500–1999 g	102	21	20.6	2.01 (0.95–4.27)		8
1000–1499 g	251	42	16.7	1.56 (0.80–3.04)		10
750–999 g	211	68	32.2	3.69 (1.94–7.04)		14.5
<750 g	242	126	52.1	8.44 (4.49–15.8)		12

Of the 994 infants with NEC, 695 were discharged alive before December 31^st^, 2014. Of these, 16 died during the follow-up period (5 died within the first month and 7 more within the first year). This corresponded to a 1-year survival of 98% (97%-99%) of NICU graduates and a 10-year survival of 97% (95%-98%).

Primary surgery was performed on 414 of the 994 NEC infants (41.6%). Furthermore, 166 infants (40.1%) underwent one or more re-operations before discharge. Of the 414 surgically treated infants, 135 were given a stoma at the primary surgery and 26 were given a stoma during a later re-operation. Fifty infants (12.1%) died on the same day or the day after surgery. At the end of follow-up, 26 infants had been given the diagnosis of short-bowel syndrome corresponding to a risk of 4.27% (95% CI 2.95–6.17) within the first 20 years of life, while 38 were admitted with ileus corresponding to a risk of 6.65% (4.69–9.44%) within 20 years. Due to few occurrences of ‘stricture or stenosis’ (n = 0) and fistula (n = 2), these were not analyzed further.

## Discussion

We confirmed the overall increased incidence of NEC during the 20-year period. Due to the large numbers coming from a national cohort over a 20-year period, the statistical significance was high, but the absolute increase in risk was small. Overall, the increase was explained by the increased survival, but. a significant GA-group/epoch interaction in the cause-specific hazard ratios suggested that the most preterm infants had an increasing risk of NEC, even when accounting for the increased survival (p = 0.06). The increased survival, however, may have occurred among the most vulnerable children (i.e. the children who would have died 20 years ago) and these children may be extra susceptible to NEC. A fact that statistical analyses cannot adjust for.

Our study was able to analyze data from an entire population over a 20-year period, thereby giving us an opportunity to determine the changes in NEC incidence for an entire country. The Danish registers are generally precise and easy to use as every citizen can be identified by their CPR-number which is used by all healthcare institutions in the country. However, Denmark is a small country, and even though we followed the entire new-born population for 20 years, we only had 994 cases of NEC. We were able to control for death as a competing outcome, thereby taking the decreased mortality into consideration.

In Denmark, the ICD-10 classification system has been used since 1994 which is why we did not study infants born before 1994. The code DP77.9– *Necrotizing enterocolitis in newborn*, *unspecified*, has been used nationally in Denmark for all cases of NEC since 1994, however, we do not know the diagnostic criteria used over the years. We assumed that the clinicians who diagnosed the infants with NEC, gave the diagnosis to infants with a Bell stage of 2 or 3 as this is standard according to international consensus, however the Danish reporting system does not provide definitions. We have previously made a single-center investigation of the validity of the NEC-diagnosis given at discharge and found a sensitivity of 0.75 and a positive predictive value of 0.57 when compared to a golden standard defined as an expert panel going through the medical records of the infants in retrospect [16]. A misclassification bias may therefore have occurred. We do not know if the validity has changed over the years, but the unchanged proportion of NEC-cases subjected to surgery and the unchanged case fatality suggest that any problem with validity would have been reasonably constant over time. Another significant weakness of our study was that we were not able to determine the exact date when the NEC diagnosis was given and therefore had to set a pseudo-date as the mid of the first specific department-admission during which NEC was registered which will affect the time-analyses.

Researchers from our neighboring country, Sweden, have performed the only other study, we are familiar with, where an entire population has been studied [5]. Their cohort consisted of all newborn infants born between 1987 to 2009. There was an overall incidence of 3.4 NEC cases per 10.000 births compared to 7.4 in our cohort. However, in Sweden the increase in incidence was larger than in Denmark rising from 2.6 NEC cases per 10,000 infants in the first epoch (1987–1992) to 5.7 in the last epoch (2005–2009) [5]. They also report decreasing mortality in all GA groups during the observation period. Unfortunately, death rate among NEC infants was not reported in the Swedish study which is why we could not determine if the higher incidence in Denmark was partly caused by a lower threshold for giving the diagnosis. The Swedish definition of NEC was from the ICD-9 system which should not be different from the ICD-10 system. Denmark and Sweden both had high rates of breastfeeding and routine use of human donor milk as well as skin-to-skin care during the periods investigated [17,18]. It may, nevertheless, be speculated that differences in nurse-to-patient ratios, education or feeding regimens may differ systematically between the countries to cause these differences, however it is beyond the scope of this article to go further into this speculation.

In January 2018, a systematic review of the incidence of NEC in high-income countries was published [19]. They reported large differences in NEC incidences between countries, ranging from 2–7% in infants born before 32 weeks of gestation. NEC mortality ranged from 21.9% to 38% which is in line with our findings.

Death rate in our cohort was almost reduced by 50% when comparing BWs 750–999 (32.2) to 1000–1499 g (16.7) and GA 23–27 (44.6) to 28–31 (17.5), highlighting the severe implications of extreme prematurity. Our data showed that there was a low but non-negligible risk of late consequences such as short-bowel syndrome or ileus during childhood, which should be of consideration when informing the parents. However, the care for infants with short-bowel syndrome has improved during the last decades and long-term quality of life in children with short-bowel syndrome was reported comparable to healthy peers [20].

In conclusion, this study underlines that NEC is still an increasing concern in the preterm population and may increase in the next years if the clinicians continue to do better in helping the most premature infants survive the first days and weeks of life. Furthermore, since increased survival did not seem to be the only cause of the increased incidence of NEC in very preterm infants, further studies should focus on this group. Focused comparison of care practices between countries that have a low incidence and a high incidence of NEC may be a way to raise hypotheses for further research.
